# P-295. Association of HIV Pre-exposure Prophylaxis (PrEP) Initiation and Persistence with Prior Visit with the PrEP Prescriber among Cisgender Women in the Bronx, NY, 2012-2023

**DOI:** 10.1093/ofid/ofaf695.516

**Published:** 2026-01-11

**Authors:** Cristal Finkenberg, Caroline E Mullis, Marla J Keller, Uriel Felsen, Jessica McWalters, Martin F Packer

**Affiliations:** Albert Einstein College of Medicine, Bronx, NY; Albert Einstein College of Medicine, Bronx, NY; Albert Einstein College of Medicine, Bronx, NY; Montefiore Medical Center, Bronx, New York; Albert Einstein College of Medicine and Montefiore Medical Center, Bronx, New York; Montefiore Hospital/Albert Einstein College of Medicine, North Adams, Massachusetts

## Abstract

**Background:**

Pre-exposure prophylaxis (PrEP) is an effective, user-controlled HIV prevention strategy, but uptake and persistence are low in cisgender women (CGW) [1-8]. Providers have a crucial role in supporting PrEP use, and a preexisting patient-provider relationship may support PrEP initiation and persistence.

**Table 1.**
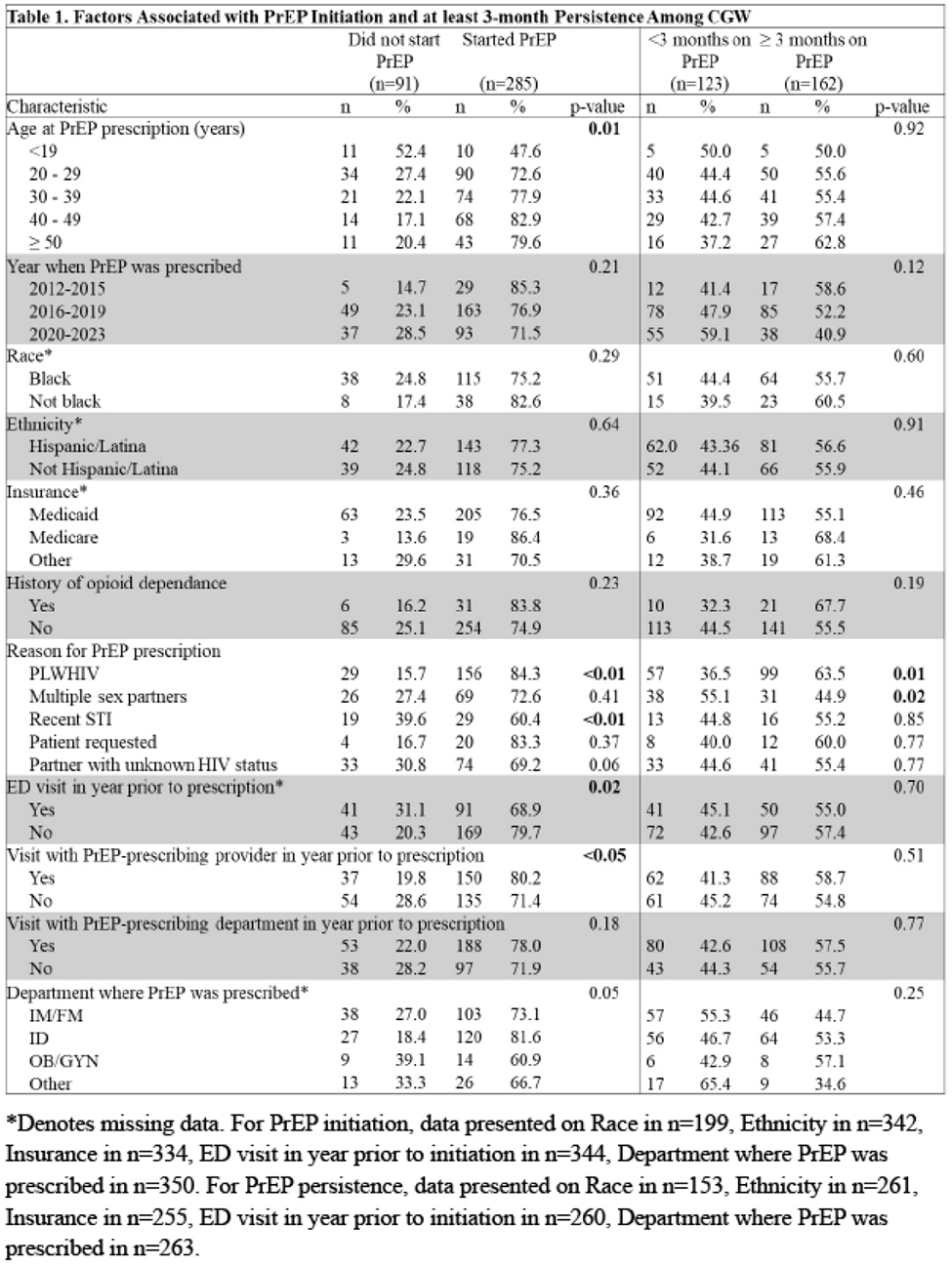
Factors Associated with PrEP Initiation and at least 3-month Persistence Among CGW Significant differences in PrEP initiation (p<0.05) were observed in age group, partner living with HIV, recent STI, ED visit in year prior to prescription. Visit with PrEP-prescribing provider was significantly associated with PrEP initiation (p<0.05) but not persistence at ≥ 3 months. CGW = cisgender women. ED = emergency department. ID = infectious disease. IM/FM = Internal medicine and family medicine. OB/GYN = obstetrics and gynecology. PLWHIV = partner living with HIV. PrEP = preexposure prophylaxis. STI = sexually transmitted infection.

**Table 2.**
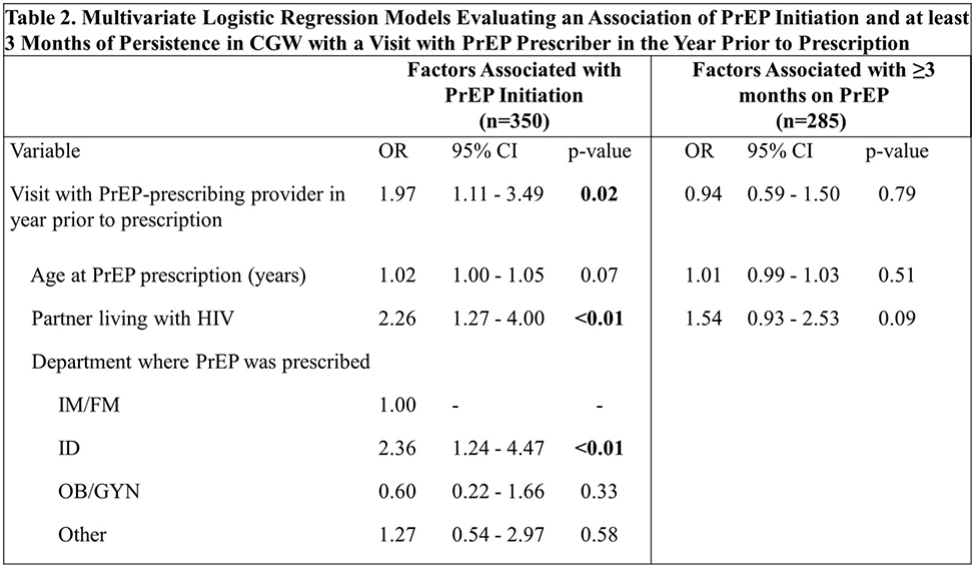
Multivariate Logistic Regression Models Evaluating an Association of PrEP Initiation and at least 3 Months of Persistence in CGW with a Visit with PrEP Prescriber in the Year Prior to Prescription PrEP initiation was significantly associated with a visit with the PrEP-prescribing provider in the year prior to prescription (p=0.02) and prescription by the ID department (p<0.01). CGW = cisgender women. CI = confidence interval. ID = infectious disease. IM/FM = internal medicine and family medicine. OB/GYN = obstetrics and gynecology. OR = odds ratio. PrEP = preexposure prophylaxis.

**Methods:**

A cohort of CGW at Montefiore Medical Center (MMC) receiving a PrEP prescription from July 2012, to July 2023 was identified from the Einstein-Rockefeller-CUNY Center for AIDS Research Clinical Cohort Database using a validated algorithm and by manual review of the electronic health record (EHR) [9]. PrEP initiation was defined as documentation of starting PrEP after prescription and PrEP persistence as documentation of continued PrEP use at 3, 6, and/or 12 months. Participants with no visit at MMC 12 months following prescription (n=60) and no documentation of PrEP initiation (n=66) were excluded. Chi-squared tests evaluated bivariate associations. Multivariate logistic regression models included age, partner HIV status and other statistically significant variables to evaluate if a visit with the PrEP prescriber the year prior to prescription was associated with PrEP initiation or persistence.

**Results:**

Of 502 CGW with PrEP prescriptions, 285 (57%) initiated PrEP, 119 (24%) continued PrEP at 3 months, 87 (17%) at 6 months and 54 (11%) at 12 months. Most of the included 376 CGW were Black (77%, 153/199) and/or Hispanic (54%, 185/342) (Table 1). A visit with the prescribing provider in the year prior was associated with 1.97 times the odds of initiating PrEP (95% CI 1.11 - 3.49, p=0.02) but not with persistence (aOR=0.94, 95% CI 0.59 - 1.50, p=0.79) (Table 2). Having a partner living with HIV was associated with 2.26 times the odds of initiation (95% CI 1.27 - 4.00, p< 0.01) but not persistence (aOR 1.54, 95% CI 0.93 - 2.53, p=0.09).

**Conclusion:**

PrEP initiation is higher among CGW who had a visit with the prescribing provider in the year prior, suggesting that knowing the prescribing provider could support PrEP uptake. Additional research is needed to better understand the patient-provider relationship dynamics to develop interventions to support PrEP use for CGW.

**Disclosures:**

All Authors: No reported disclosures

